# Stress among Mansoura (Egypt) baccalaureate nursing students

**DOI:** 10.4314/pamj.v8i1.71083

**Published:** 2011-03-16

**Authors:** Mostafa Amr, Abdel-Hady El-Gilany, Hanan El-Moafee, Lamea Salama, Cristóbal Jimenez

**Affiliations:** 1 Departments of Psychiatry and Community Medicine, College of Medicine, Mansoura University, Mansoura, Egypt; 2 Department of Community Nursing, College of Nursing, Mansoura University, Mansoura Egypt; 3 Department of Nursing, School of Nursing University of Córdoba, Andalucía, Spain

**Keywords:** Nursing students, stress, Depression, Anxiety, Arab culture, Egypt

## Abstract

**Background:**

Over the last years, details regarding levels of stress and sources of stress have emerged in studies of nursing students in Western population To date, there only few similar reports on clinical stress, anxiety, depression among the Arab population .This study was conducted to examine the level of perceived stress among baccalaureate Mansoura nursing students and to highlight the possible predicting factors.

**Methods:**

In this cross- sectional study, Data were obtained from 373 students using a self-administered questionnaire, including questions on sociodemographics, list of possible stressors, perceived stress, physical wellbeing factors, anxiety and depressive symptoms.

**Results:**

Prevalence of high stress level, anxiety and depression were 40.2%, 46.6% and 27.9%, respectively. On average each student reported a mean of 4.6 stressors and academic pressures were the most frequent stressors .In regression analysis the number of stressors and global sickness index score were predictors of high stress level.

**Conclusion:**

These findings call for introduction of stress management programs and psychiatric care into nursing health services of the University.

## Background

Stress has been identified as a 20th century disease and has been viewed as a complex and dynamic transaction between individuals and their environments [1]. Stressors can be broadly defined as situations or events that have the potential to affect health outcomes [2]. Cognitive appraisal, defined as the individual interpretation of a potential stressor, as a mediating process, can influence the impact of stress on health [3]. People who view the events as threatening (i.e., cannot employ adaptive coping skills) are more prone to cognitive deficits (for example, attention and concentration difficulties), physical illness, decreased life satisfaction [4-7], neuroticism [8], poor health behaviors and impaired academic performance [9].

The faculty of nursing, Mansoura University was founded in 2000. The 4-year undergraduate curriculum in Mansoura is designed to ensure that the theoretical and clinical practice requirements identified by the Nursing sector Committee of the Supreme Council of Egyptian Universities are met at appropriate levels throughout the 4 years of the program. During the clinical practicum, students are assigned to various clinical specialties in hospitals and medical centers of the university to gain clinical experience. Their clinical knowledge, skills, problem solving ability, and professional attitudes have to be assessed in each clinical practicum course [10]. Levels of stress and sources of stress have been reported in studies of nursing students in Western population [1,11-14] However, there has been limited research on clinical stress, anxiety, depression among the Arab population [15,16]. No study has investigated the perception of stress among Egyptian baccalaureate nursing students. This study was conducted to examine such students’ stress. The research questions of the study were the following: 1) what is the level of stress perceived by baccalaureate nursing students? 2) What type of stressors are commonly experienced by the students? 3) What is the level of anxiety and depression perceived by students? 4) What factors are associated with the frequency of stress experienced by the students?

## Methods

This was a cross-sectional descriptive study.

### Setting and sample

The study was conducted on nursing students of Mansoura College of Nursing during March (after mid-year vacation) of the academic year of 2008/2009 G.

After literature review, a specially designed questionnaire was in English was used as the tool for data collection. This was pilot tested on a sample of 40 students (10 from each educational year), over a one-week period (not included in the full-scale study). An interview was conducted after approval of the students. The questionnaire was modified accordingly in its final form e.g. rephrasing of some question and adding explanatory notes. This pilot study revealed that about 35% of students suffer severe stress. Finally the questionnaire was approved by the college authority as there is no formal research ethics committee. Sample size was calculated using Epiinfo^®^ version 6.02. According to students’ affairs administration, the total number of registered nursing students was 1627 (all females)in the four years. From the pilot study it is expected that at least 35% of students suffer severe stress. With the worst acceptable level 32%, the sample needed for the study was estimated to be at least 350 students at 95% confidence level. First students were stratified into the different academic years (first to fourth). From each year Students were selected through systemic sampling technique (every 4th student) using the master list of students’ academic numbers. A total of 402 questionnaires were distributed and 381 were returned.Of these 8 were excluded due to incomplete or missed data. Thus 373 questionnaires were analyzed with a response rate of 92.8%.

### Data collection

Participants completed an anonymous self-administered questionnaire two months before examination to avoid its possible direct stressful effect. The questionnaire covered socio-demographic factors, grade of the previous year, presence of stressors if any that had occurred during the past twelve months; Perceived Stress Scale (PSS), assessment of physical well-being factors, hospital anxiety and depression scale, neuroticism and extraversion subscales of Eysenck personality questionnaire, including fifteen potential sources of stress (stressors) were included. Students were asked to indicate the specific stressors, if any, affecting them. Perceived stress was measured by a previously validated 14-item Perceived Stress Scale (PSS) likert type,.The PSS has internal consistency of 0.85 (Cronbach ∝ coefficient) and test-retest reliability during a short retest interval (several days) of 0.85 [17]. The Arabic version was tested among a sample of US Arab immigrants [18]. The PSS does is not linked to appraisal of a particular situation as it is sensitive to the non-occurrence of events as well as to ongoing life circumstances. High scores on the PSS mean greater stress levels.The stress score was stratified into “no”, “mild”, “moderate” (less than the first, second and third quartiles, respectively) stress (All merged under “low level” stress, or severe (equal to or above the fourth quartile) labeled as high level stress.

A self-report questionnaire for assessing physical well-being factors, designed by Hojat et al.[19] included 15 health problems: a) somatic symptoms of stress including questions about skin rash, back pain, allergies, infectious diseases, frequent colds and generalized body pain; b) agitation symptoms e.g. sleep problems, headache, nausea, lack of appetite; c) eating/drinking and smoking problems; and d) chronic illness and health problems interfering with daily activities. The global sickness index was based on an average score obtained from all health problems listed in the questionnaire. The Cronbach ∝ coefficients for the four physical well-being factors were in the 0.90s.

The hospital anxiety and depression scale (HAD) [20] was used to measure subjective anxiety and depression where a score of 12 or more for either the anxiety or the depression components denotes possible anxiety or depression. This cut off point has a sensitivity of 0.89 and specificity of 0.75 [21]. The Arabic version of the HAD scale was validated by El-Rufaie and Absood [22]. The overall Cronbach ∝ measures of internal consistency were 0.78 and 0.87 for anxiety and depression, respectively.

### Procedure

The questionnaire used in the study was administered in classrooms under the guidance of the researchers. Students were briefed about the study, encouraged to participate and motivated to express their experiences. The students give fully informed verbal consent to participate. It was emphasized that all data collected was strictly confidential. Efforts were made to minimize under-reporting, strongly emphasizing to the student that the questionnaire was anonymous and that the data would be used for scientific purposes only. The questionnaires were distributed and recollected in the same setting.

### Data Processing and analysis

Data was analyzed using SPSS (Statistical Package for Social Sciences) version 11. In quantitative data unpaired student’s t-test was used for group comparison. In categorical data Chi-squared test was used for comparison between groups. Odds ratio and 95% confidence interval was calculated. Significant factors predicting stress on univariate analysis were entered into multivariate logistic regression analysis. P<0.05 was considered statistically significant.

## Results

The study included 273 Baccalaureate Nursing Students. Their age ranged from 17-22 years with a mean of 18.8±1.2, 68 % of the sample was from rural areas. Family income was reported to be satisfactory in 78.2 % of students and a family size of more than 5 persons was reported by 55.7% of students. 77.5% of fathers and 75.9 % of mothers of the respondents were of secondary and above secondary education level. More than two thirds of fathers were working as professionals (63.8%) and 59.2% of mothers were housewives. Mild to moderate stress level (Low stress) and severe stress (High stress) were encountered in 59.8 and 40.2% of the students respectively. Clinical anxiety was reported in nearly half of the sample (46.6%) and depression in 104 students (27.9%).

Stressors were reported by 97.3% of students and the number of stressors reported by students ranged from 0 to 13, with a mean of 4.6±2.5. The reported rate for the occurrence of stressful events ranged from a low of (9%) for the death of a family member to a high of 82.6% for "fear of future". The five most frequently reported stressors were fear of future, self reported anxiety and depression, increased class workload, accommodation problems and congested classrooms (Table 1). Regarding the PSS, the mean overall score was 28.6±6.7(15-89), low and high stress groups were 27±4.9(7-32) and 38 ±4.6(33.55). Table 2 Shows that the prevalence of high level of stress, anxiety and depression were 40.2%, 46.6% and 27.9%; respectively. The overall mean of global sickness index was 28.6±6.7(15-89).

There were significant differences in the sociodemographic data of those with high and low stress level such as family residence (p= 0.016), father’s education (p=0.015), father’s work (p=0.022) and grade of previous year (p= 0.031).The high stress group had a significant trend for living in rural areas, their fathers were less frequently professional, had lower school education and grade in the previous year (Table 3).

Multivariate logistic regression analysis (Table 4) showed that that the number of stressors and Global Sickness Index score (GSI) were independent predictors of high stress level (p=0.000 and p=0.04 respectively).

## Discussion

Our study showed that 40.2% of nursing students who reported high stress (40.2%) which was higher than other studies using different distress measures in both developed and developing countries. Papazisis et al. [14] reported that 71.8% of nursing students in Greece perceived stress, most of them in mild levels (31.8%). About 12.4% reported very high levels of stress. Pryjmachuk and Richards [13] found that that the overall General Health Questionnaire caseness in pre-registration nursing students was around one-third. Arafa et al. [23] revealed that 21.67% of nurses recorded moderate to severe psychological distress on the General Health questionnaire (GHQ-30 items). This higher prevelance of stress in our sample could be interpreted as follows:

Firstly, In Egypt, the current education policy allows an increasing number of admitted college students.The performance and quality of higher education is severely compromised. Traditionally, most students spend the majority of their school careers in passive learning environments in which faculty are disseminators of information, and students are required to memorize information. The system doesn’t foster the development of synthesising, problem solving and creative thinking abilities that impair the clinical skill training [24].

Second, in Egypt there are three types of nurses: college graduates, technical institute graduates and nursing school graduates, also known as diploma nurses. The first two types of nurses comprise four and two per cent of the Egyptian nursing staff respectively, while diploma nurses make up the remaining 94 per cent [25]. Recent reforms in the health sector eliminated the high school nursing by 2009, allowing a gradual replacement of diploma nurses with baccalaureate graduates,thus bringing about a change in the already established role of college graduates as staff nurses having the leadership and prestigious positions in the hospital environment to assistant practical nurses who only obey the orders of the junior doctors and senior diploma nurses.

Third, these future nurses are expecting financial hardships after graduation due to low salaries which might reflect on their future social and family life [26]. Nurses are often blamed for errors, and are generally treated as scapegoats by other members of the medical profession [27].

Fourth, families in the Arab world are undergoing major changes as new patterns of marriage emerge across the region [28]. Early marriage is no longer the standard it once was in Arab countries: the average age at marriage for both men and women is generally rising, and more Arab women particularly who are college graduates are staying single longer or not marrying at all [29].

Finally, the poor reputation of the nursing profession in Egypt because of the involvement of the nurse (female) and the doctor (male) on the one hand or the nurse and the patient on the other hand [27].

Stressors identified by the students in this study such as self reported anxiety and depression, increased class workload and, financial and relationship problems are similar to what been described in previous studies of similar student groups [19,30,31].

Despite some similarity in the types of stressors identified, perceived stressors such as worry of students towards their future, accommodation problems and overcrowded classrooms appear unique to this setting. Having to cope in a crammed classes and hostels would be extremely stressful in a course with a heavy workload requiring increased study time. The observations made by the students have been substantiated by college authorities who carried a program to motivate students’ active learning through applying problem-based learning in the third year of an undergraduate nursing program among some groups of students who enrolled in nursing administration course. The students in the PBL group gained more knowledge and were more motivated for learning than those in the lecture group [32].

On logistic regression analysis, High Stress was positively associated with number of stressors, and the global sickness index. A meta-analysis of 40 students on psychological distress among U.S. and Canadian college students explored the relationship between level of perceived stress and student distress [33]. Okasha et al. [34] concluded that most cases of psychological distress among Egyptian college students were reactions to either maturational or environmental stresses rather than endogenous factors. It is possible that medical students find medical education stressful with a high stress level reported along with a higher level of related psychosomatic activity and increased mood disturbances [17]. Liu and colleagues [35] showed that poor health status, test pressure, conflict with classmates and personality trait of introversion were independently associated with the presence of anxiety. Cohen and Williamson [36] added that stress as measured by the PSS would be moderately correlated with the number of stressors.

The limitations of this study are that the findings are based on self-reported information provided by students and thus some potential for reporting bias may have occurred because of respondents’ interpretation of the questions or desire to report their emotions in a certain way or simply because of inaccuracies of responses. The study takes place at one point in time which will limit the ability to generalize the findings to other time periods; this is referred to as a threat to temporal validity. In addition, the study took place at only one college, which will affect the generalizability to other institutions.

## Conclusion

It is clear that the Egyptian student nurses surveyed, were exposed to a variety of academic, personal and environmental stressors. Academic pressures and the effect on social life and means for sustenance are key areas for intervention. The importance of small classes, active method of learning provision of equipment for practical work, improving accommodation should be emphasized. Given the detrimental effects of stress on health and academic performance, college administrators should consider incorporating stress management training into orientation activities for nursing students. Other approaches may be the use of stress management, assertiveness skills, time management and counseling sessions, may be effective in reducing stress experienced by nursing students. More studies need to be considered at a multi-center level using more informative sociodemographic, psychosocial and institutional variables in order to confirm the present findings and to enlighten corrective interventions.

## Competing interests

The authors declared they have no competing interests. Also there are no sources of funding.

## Authors contribution

MA: Study concept, design, Statistical analysis, data discussion manuscript writing, and editing. AG: Study concept, design of the questionnaires, Statistical analysis, manuscript writing and review. HM, LS: Female researchers who made awareness of the students, Data collection and manuscript writing and review. CJ: helped in manuscript review and editing

## Acknowledgments

The authors would like to thank all the nursing students at the College of Nursing, Mansoura University for their sincere cooperation.

## Figures and Tables

**Table 1:** Prevalence of different stressors among Mansoura (Egypt) baccalaureate nursing students (n=373)

	N (%)
**No stressors**	10(2.7)
Number of stressors X± SD (Min – Max) Stressors*	4.6 ± 2.5 (0–13)
**Relationship issues**	
Relationship problems with parents	54(14.5)
Problems with the opposite gender	85(22.8)
Trouble with coursemates	80(21.4)
**Personal troubles**	
Personal illness or injury	56(15.0)
Death of a family member	37(9.0)
Change of a family member’s health	83(22.3)
Financial problems	115(30.8)
Self-reported anxiety and/or depression	237(63.5)
**Academic pressures**	
Congested classroms	174(46.6)
Increased class workload	223(59.8)
Inconsiderate and insensitive instructors.	72(19.3)
Fear of future	308(82.6)
**Environmental problems**	
Accomodation problems**	203(54.4)
Close contact with serious diseases and illness	80(21.4)
Time limitation for sports and hoppies	156(41.8)

* Categories are not mutually exclusive

** e.g. overcrowed accomodation, noisy livining environement, transportation problems.

**Table 2:** Overall prevalence of stress, anxiety and depression stressors among Mansoura (Egypt) baccalaureate nursing students

	N (%)	95% CI
High stress	150(40.2)	35.4–45.3
Anxiety	174(46.6)	41.6–51.7
Depression	104(27.9)	23.6–32.6

CI = Confidence interval

**Table 3:** Level of stress according to sociodemographic and academic characteristics of stressors of Mansoura (Egypt) baccalaureate nursing students

	Stress level			Significance
	Low N (%)	High N (%)	Total N (%)	
**Overall Family residence**	223(59.8)	150(40.2)	373(100)	
Urban	63(52.9)	56(47.1)	119(31.9)	c^2^=8.2,
Rural	160(63.0)	94(37.0)	254(68)	P=0.016
**Student’s residence**				
With family	174(59.8)	117(40.2)	291(78)	
Away from the family	49(59.8)	33(40.2)	82(22)	
**Family income**				
Unsatisfactory	42(51.9)	39(48.1)	81(21.8)	c^2^=2.7,
Satisfactory	181(62.0)	111(38.0)	292(78.2)	P=0.1
**Family size**				
Up to 5	104(62.7)	62(37.3)	166(44.5)	c^2^=1.02,
> 5	119(57.5)	88(42.5)	207(55.4)	P=0.3
**Father’s education**				
Less than secondary	54(64.3)	30(35.7)	84(22.5)	c^2^=8.4,
Secondary	87(51.8)	81(48.2)	168(45)	P=0.015
Above secondary	82(67.8)	39(32.2)	121(32.5)	
**Father’s work**				
Unemployed	19(50.0)	19(50.0)	38(10.1)	c^2^=7.6,
Professional/semiprof	135(56.7)	103(43.3)	238(63.8)	P=0.022
Others*	69(71.1)	28(28.9)	97(25.3)	
**Mother’s education**				
Less than secondary	57(63.3)	33(36.7)	90(24.1)	c^2^=1.5,
Secondary	118(57.0)	89(43.0)	207(55.5)	P=0.5
Above secondary	48(63.2)	28(36.8)	76(20.4)	
**Mother’s work**				
Working	87(57.6)	64(42.4)	151(40.8)	c^2^=0.5,
Housewives	136(61.3)	86(38.7)	222(59.2)	P=0.5
**Academic year**				
First	55(55.0)	45(45.0)	100(26.8)	c^2^=3.7,
Second	64(56.1)	50(43.9)	114(30.6)	P=0.3
Third	59(65.6)	31(34.4)	90(24.1)	
Four	45(65.2)	24(34.8)	69(18.5)	
**Grade of previous year**				
Excellent	91(54.8)	75(45.2)	166(44.5)	c^2^=8.9,
Very good	98(69.0)	44(31.0)	142(38.1)	P=0.031
Good	11(45.8)	13(54.2)	24(6.4)	
Others**	23(56.1)	18(43.9)	41(11.0)	

* Others include : private business, farmers, manual workers, trades

** Others include : pass and failed

**Table 4:** Multivariate analysis using a stepwise logistic regression model

Predictor	High stress level		
	b	P	OR(95% CI)
**Global sickness index:(continuous)**	0.04	0.04	1.01 (1.01–1.1)
**Number of stressors: (continuous)**	0.2	0.000	1.2 (1.1–1.3)
**Constant**	−2.4		
**Percent correctly predicted**	64.3%		
**Model c^2^**	41.7, P=0.000		

OR = Odds ratio, CI = Confidence Interval, r = reference group
